# Reference Materials for Calibration of Analytical Biases in Quantification of DNA Methylation

**DOI:** 10.1371/journal.pone.0137006

**Published:** 2015-09-14

**Authors:** Hannah Yu, Yoonsoo Hahn, Inchul Yang

**Affiliations:** 1 Center for Bioanalysis, Division of Metrology for Quality of Life, Korea Research Institute of Standards and Science, Daejeon, 305–340, Republic of Korea; 2 Bio-analytical Science, University of Science and Technology, Daejeon, 305–340, Republic of Korea; 3 Department of Life Science, Chung-Ang University, Seoul, 156–756, Republic of Korea; University of Bonn, Institut of experimental hematology and transfusion medicine, GERMANY

## Abstract

Most contemporary methods for the quantification of DNA methylation employ bisulfite conversion and PCR amplification. However, many reports have indicated that bisulfite-mediated PCR methodologies can result in inaccurate measurements of DNA methylation owing to amplification biases. To calibrate analytical biases in quantification of gene methylation, especially those that arise during PCR, we utilized reference materials that represent exact bisulfite-converted sequences with 0% and 100% methylation status of specific genes. After determining relative quantities using qPCR, pairs of plasmids were gravimetrically mixed to generate working standards with predefined DNA methylation levels at 10% intervals in terms of mole fractions. The working standards were used as controls to optimize the experimental conditions and also as calibration standards in melting-based and sequencing-based analyses of DNA methylation. Use of the reference materials enabled precise characterization and proper calibration of various biases during PCR and subsequent methylation measurement processes, resulting in accurate measurements.

## Introduction

Cytosine methylation at CpG sites plays pivotal roles in gene expression regulation and the maintenance of cellular functions in vertebrates [1,2]. Dysregulation of DNA methylation can result in a variety of diseases including cancers [3]. Many reports have indicated that altered DNA methylation is correlated with various cancers and the detection of specific aberrant methylation provides diagnostic and prognostic information about those diseases [4]. However, the sensitivity and specificity of DNA methylation-based diagnostics are often variable depending on measurement platforms and operational protocols [5,6]. Inconsistent receiver operating characteristic (ROC) data resulting from variation in measurement sensitivity and specificity limits the effectiveness of DNA methylation-based diagnostics [7,8]. Researchers have indicated the need to establish a standard system for DNA methylation by which measurement performances in research fields and clinical laboratories can be evaluated and calibrated. An ideal standard system for measurement of DNA methylation will involve accurate measurement methods and relevant reference materials. Reference materials with accurately assigned values could be used to optimize analytical procedures and calibrate biases in specific measurement practices. Accurate and consistent measurements of DNA methylation achieved based on the standard system will facilitate more accurate, discriminative, and consistent diagnoses of various DNA methylation-related diseases.

The majority of quantitative DNA methylation analyses employ bisulfite conversion and PCR. Bisulfite conversion transforms DNA methylation information into sequence information, i.e., unmethylated cytosine is converted to uracil and methylated cytosine remains as cytosine. The transformed sequence information is maintained throughout PCR and quantitatively analyzed by various post-PCR measurement methods. Post-PCR measurement methods include a variety of approaches such as clonal sequencing [9], restriction enzyme digestion [10], quantitative PCR (qPCR) [11], high-resolution melting analysis [12–14], mass spectrometry [15], pyrosequencing [16], and next-generation sequencing [17]. Since most measurement methods involve bisulfite conversion and PCR, biases and variation introduced during those processes affect the final estimates of DNA methylation, irrespective of post-PCR analytical platform. Accordingly, bisulfite conversion and PCR processes are major targets for optimization and technical improvements to achieve more consistent and accurate measurements of DNA methylation. Although incomplete bisulfite conversion affects the accuracy and fidelity of DNA methylation analyses, effects of the conversion process can be minimized by use of improved commercial kits and optimized protocols [18]. On the contrary, biases associated with PCR processes were not efficiently controlled and remains still problematic in many studies [19–21]. Notable approaches to deal with PCR biases in DNA methylation analysis include digital PCRs in which digitized amplicons from single template molecules could be obtained and analyzed [22,23]. Despite the distinct advantages of DNA methylation analyses based on digital PCRs, instruments for digital PCR are relatively expensive and not easily accessible at present.

PCR-driven biases are highly complicated and depend on various experimental parameters such as sequence composition, combination of primers, Taq polymerase and annealing temperature [21,24–26]. Due to the complicated nature of PCR-driven biases, it is not easy to precisely predict and properly control such biases. To minimize deteriorating effects from PCR, it is necessary to characterize PCR-driven biases in detail. An elaborate characterization of PCR biases will help optimization of experimental procedures and parameters for minimization of PCR biases, which will improve accuracy and consistency of measurements. Use of reference materials of which DNA methylation values were accurately determined could be a solution not only for precise verification but also for appropriate calibration of PCR biases in DNA methylation analyses. Researchers use either commercial or in-house controls with low and high methylation to verify biases [27,28]. However, DNA methylation levels of those controls were not accurately assigned nor extensively validated with respect to specific genes. Use of controls with gene methylation values that lack metrological validity can lead to incorrect identification of PCR biases. In addition, accurate measurement of DNA concentration is very important for preparation of intermediate level controls by mixing the low- and high-methylation controls. Inaccurate measurements of DNA concentrations of starting samples could lead to inaccurate assignments of reference values for the mixed intermediate level controls. Furthermore, measured total DNA concentrations of the low and high controls need to be converted to relative quantities with respect to specific target genes, and this is not generally possible for commercial controls. Based on this consideration, pairs of reference materials of which DNA methylation levels and relative concentrations are accurately known with respect to specific genes could provide improved solutions for correct verification and calibration of PCR biases in the quantification of DNA methylation.

In an effort to develop reference materials for measurement of DNA methylation status of specific genes, we suggest use of artificial plasmid constructs with pre-defined sequences that represent exactly 0%- (M0) and 100%-methylation (M100) of genes. Since most DNA methylation analyses involve bisulfite conversion reactions, the proposed reference materials were designed to represent bisulfite-converted sequences. The materials were aimed to be used for verification and calibration of biases during PCR and subsequent post-PCR processes. In this paper, we present a proof-of-concept for utilization of such template-type reference materials for DNA methylation. The template-type reference materials for three model genes were prepared by chemical synthesis and used to examine and calibrate various PCR biases in DNA methylation measurements.

## Material and Methods

### Preparation of M0 and M100 plasmid constructs

Plasmid constructs containing bisulfite-converted sequences that represent exactly 0%- (M0) and 100%-methylation (M100) of the *INK4A (P14)*, *CDKN2A (P16)*, and *MLH1* genes were synthesized and purified using high-performance liquid chromatography through a commercial service (Bioneer, Daejeon, Korea). The sequence information for the synthesized inserts is presented in S1 Data. Sequence-verified plasmid DNA (3–4 μg) was linearized by PvuII (New England Biolabs) and purified by ultrafiltration (Ultracel-30K; Millipore). Linearized plasmids were used as stock materials for preparation of working standards.

### Preparation of working standards

**Fig 1:**
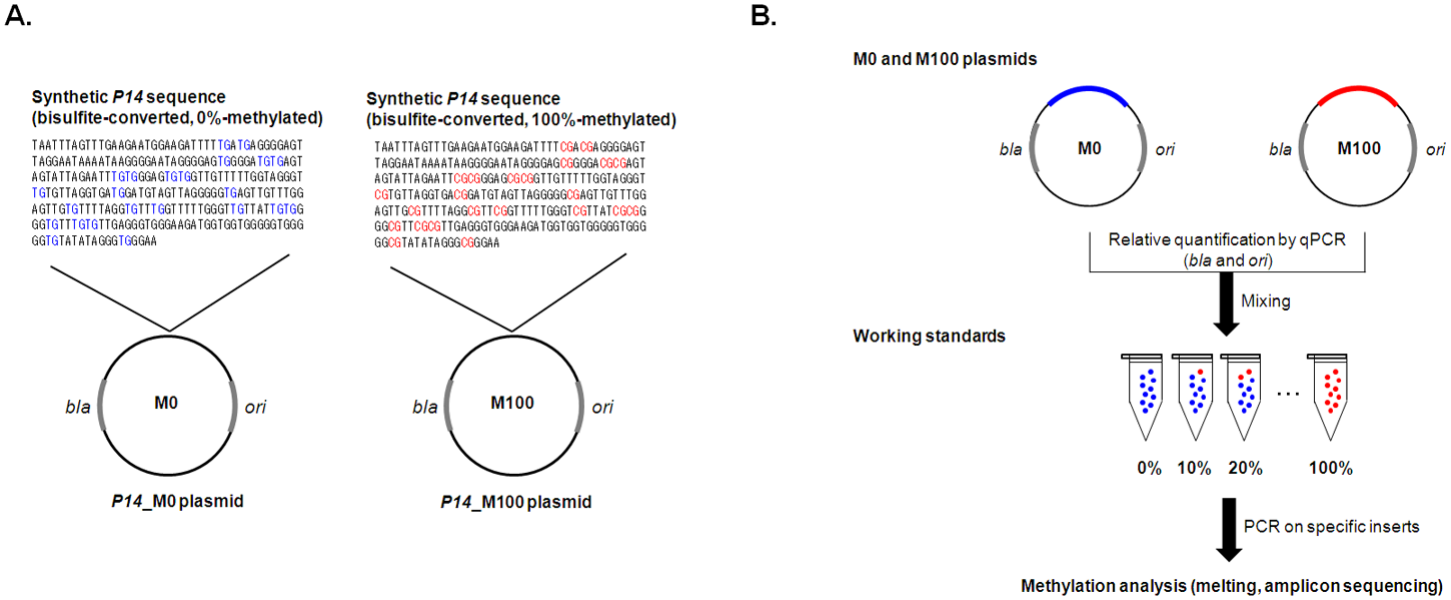
Concept of the synthetic reference materials for DNA methylation. (**A**) Structures of a plasmid pair each of which harbors bisulfite-converted sequence of either 0%—(M0) or 100%- (M100) methylation status of a gene. A plasmid construct is composed of a common backbone (pGEM) and a synthetic insert which represents bisulfite-converted sequence of either 0%- or 100%- methylation status of gene. (**B**) Use of the reference materials as working standards in DNA methylation analysis. qPCRs targeting common backbone sequences such as *bla* and *ori* regions were performed to determine relative quantities of the plasmid pair of a gene.

The reference plasmid constructs in this study are comprised of common pGEM plasmid backbones and synthetic sequences that are bisulfite-converted sequences of 0%- and 100%-methylated genes (Fig 1A). The M0 and M100 plasmids share a common backbone originating from pGEM. To determine relative quantities of the M0 and M100 plasmid pair, qPCRs targeting *bla* and *ori* regions in the common backbone were performed. Primers for qPCR are described in S1 Table. qPCR was performed using TaqMan Universal Master Mix II (Life Technologies) on a StepOnePus Real-Time PCR system (Life Technologies). Standard two-step thermal cycling conditions (95°C for 15 seconds and 60°C for 60 seconds) were employed for qPCR. The qPCR results obtained from four independent primer combinations were averaged to estimate the relative quantities of M0 and M100 plasmid pairs for each gene. After determining the relative quantities, M0 and M100 plasmids were gravimetrically mixed to prepare working standards with methylation levels from 0% to 100% at 10% intervals in terms of mole fraction. DNA methylation levels of the prepared working standards were re-assigned based on the qPCR results and weighing records.

### In-lab preparation of unknown samples

For in-lab preparation of unknown samples, the promoter regions of three target genes were amplified from sequence-proven plasmid clones. Primers for the amplification of promoter sequences are listed in S2 Table. Methylated samples were generated by repeated *in vitro* methylation of the unmethylated PCR products using *M*.*Sss* I CpG methylase (Zymo Research) for three rounds. Levels of CpG methylation of the *in vitro* methylated DNA were measured by dNMP analyses in capillary electrophoresis (CE) [29]. Equal volumes of the unmethylated and *in vitro* methylated PCR products for each gene were then mixed to generate in-lab unknown samples. Actual DNA methylation levels of the prepared in-lab unknown samples were measured by dNMP analysis in CE. Fragmented (500 bp to 3 kbp) calf thymus genomic DNA was added as matrix DNA to the in-lab unknown samples. Copies of exogenous genes in the in-lab unknown samples were adjusted to be approximately 1,000-times higher than copies of endogenous counterparts of the matrix DNA.

### Commercial DNA methylation control

Pairs of low-methylated and high-methylated DNA controls were purchased from Epigen Dx and Qiagen. Controls from Epigen Dx were used as templates for PCR after bisulfite treatment while controls from Qiagen were directly used without bisulfite treatment.

### PCR and melting analysis

Most real-time PCRs were performed using 2× SYBR HotStart Ex Taq Premix (Takara) on a StepOnePlus Real-Time PCR instrument with the following thermal cycling conditions: 94°C for 30 seconds, 58°C for 30 seconds, and 72°C for 30 seconds. Primers for the amplification of *P14*, *P16*, and *MLH1* from bisulfite-converted templates are listed in S3 Table. PCR primers were designed using the public open software, MethPrimer (http://www.urogene.org/cgi-bin/methprimer/methprimer.cgi). Amplicons were directly subjected to melting analyses without purification after PCR. Melting analysis was performed on exponential amplicons from 18 PCR cycles. Melting analyses were performed with 0.3°C stepwise increments of temperatures from 60°C to 95°C. Raw melting profiles exhibiting different fluorescence intensities and background levels among samples were normalized to the same scale to facilitate efficient comparisons of the results. Normalization was performed using two distinct processes, ‘blanking’ to make background levels at the end of melting the same and ‘normalization’ to make the fluorescence intensities at the start of melting the same for the set of data to be compared [30]. After normalization, areas under each melting curve were calculated by summation of the fluorescence values within the melting range. Then, the areas represent the relative quantities of M0 to M100 amplicons in samples [31]. DNA methylation levels of working standards and unknown samples as determined by the integrated fluorescence intensities were plotted against the preparation values to derive a calibration curve. The methylation values of unknown samples were calculated by linear regression based on the two closest points encompassing the unknown value in the standard curve.

### NGS analysis

NGS libraries were prepared using the NEBNext Ultra DNA Library Prep Kit for Illumina (New England Biolabs) following the manufacturer’s instructions starting with 100 ng to 1 μg of purified amplicons. Since the sequences of amplicons for *P14*, *P16*, and *MLH1* were different and could be easily discriminated in the sequencing results, three amplicons were mixed together and processed as a set. To quantify the library DNA, qPCR was performed using the 2× KAPA SYBR Fast Universal qPCR Kit (KAPA Biosystems). Sequencing was conducted on a MiSeq platform (Illumina) resulting in paired-end reads of 150 bp.

### Bioinformatics

The methylation-reference sequences representing bisulfite-converted target sequences of M0 and M100 were constructed and converted to a BLAST-searchable database. For each of NGS sequence sets, BLASTN search was performed against the methylation-reference sequence database using NGS reads as queries. The command line was: “blastn-query NGS-db REFERENCE-outfmt 6-evalue 1e-5-out OUTPUT”. The BLASTN output files were parsed using an *ad hoc* Perl script to collect best-scoring matches for each amplicon read. A read was assigned to a methylated or unmethylated target when the read matched a unique reference sequence, otherwise the read was discarded. For each target, the numbers of amplicons assigned to the methylated or unmethylated reference sequence were counted.

## Results and Discussion

The use of proper reference materials may allow verification and calibration of various analytical biases in measurements of biological properties, and therefore will facilitate accurate and reliable measurements. In this study, we developed reference materials to examine and calibrate PCR biases in DNA methylation analyses. We suggest utilization of a pair of artificial reference DNA constructs that comprise common pGEM plasmid backbones and synthetic sequences representing bisulfite-converted sequences of 0%- and 100%-methylated genes (Fig 1A). The 0%-methylation reference material (M0) was designed to have TpGs at all CpG positions, while the 100%-methylation material (M100) have CpGs at the same positions. It should be noted that our strategy is concerning only about the CpG methylation but not the non-CpG methylation which was reported to be an important source for measurement biases of DNA methylation [32]. If we take the non-CpG methylation into account, a large number of different synthetic DNAs will be required to represent all possible combination of CpG and non-CpG methylation states. Therefore, we practically focused only on CpG methylation in this study.

**Fig 2:**
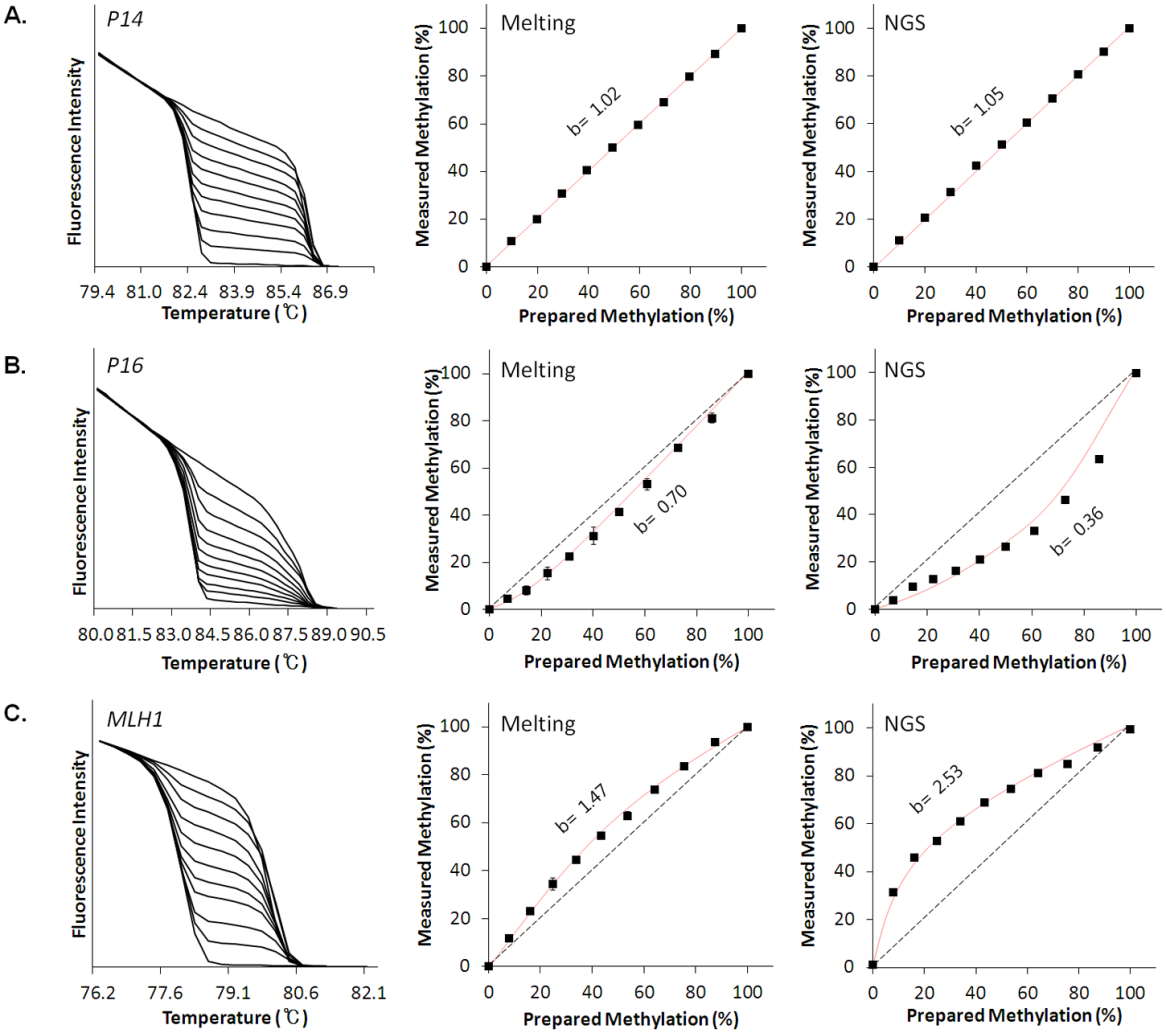
Gene-dependent PCR biases in DNA methylation analyses. Normalized melting profiles (left), standard curves from melting analyses (center) and standard curves from NGS analyses (right) from analysis of working standards of *P14* (**A**), *P16* (**B**) and *MLH1* (**C**) genes. Standard curves for NGS analyses were plotted based on the read counts for unmethylated and methylated DNA from amplicon sequencing as provided in the S5 Table. No PCR bias for *P14*, preferential amplification of unmethylated templates for *P16* and preferential amplification of methylated templates for *MLH1* were consistently observed from both melting- and NGS-based analyses. Bias values (b) were calculated based on a previously described equation [19].

Three pairs of M0 and M100 plasmid reference materials for the *P14*, *P16*, and *MLH1* genes were prepared and tested as a proof-of-concept in this study. After verifying the correctness of insert sequences, relative quantities of the plasmid pairs were quantified by performing qPCR (Fig 1B). qPCRs targeting four different loci around *bla* and *ori* regions in the common plasmid backbone were performed to determine the relative quantities of the plasmids (S4 Table). Then, the quantified M0 and M100 plasmid pairs were gravimetrically mixed to prepare working standards with pre-defined methylation levels from 0% to 100% at 10% intervals in terms of mole fraction. Working standards with smaller intervals will enable the discrimination and calibration of PCR biases with higher resolution and more accurate quantification of DNA methylation. The accuracy and measurement uncertainty of values obtained using the current calibration strategy are affected by two major factors, i.e., the scattering of data in repeated measurements and the reliability of pre-assigned values for the working standards. Additionally, the measurement uncertainty of pre-assigned values for working standards depends on three factors, i.e., the sequence purity of starting plasmid constructs, the accuracy of relative quantities of reference plasmid pairs determined by qPCR, and the fidelity of the preparation procedure for working standards by gravimetric mixing. Above all, qPCR-based assignment of plasmid quantities was the most critical source of uncertainty, explaining 3–4.3% variations among estimates from four independent qPCR experiments with different target loci (S4 Table). The effects of other sources of uncertainty for the working standards, such as plasmid purity, fidelity of the gravimetric mixing procedure, and homogeneity of working standards, were considered negligible. The purpose of the current calibration strategy was to utilize working standards with pre-defined DNA methylation values not only for precise examination of biases, but also for calibration of the biases, which will facilitate accurate measurements of DNA methylation. We expect that these template-type reference materials and subsequent working standards could be universally applied as external calibrators independent of analytical platform, such as melting-based, NGS-based, and pyrosequencing-based analyses, assuming the processes involve bisulfite treatment and PCR. For verification of PCR-driven biases, melting analyses were performed for working standards of *P14*, *P16*, and *MLH1*. Melting analyses were performed on amplicons from exponential amplification phases (18 cycles). Distinct PCR-driven biases in the melting-based DNA methylation analysis are shown in Fig 2. No PCR bias was observed in melting profiles of *P14* amplicons (Fig 2A). Accordingly, the standard curve was linear. On the contrary, a slightly downward parabolic standard curve was obtained for *P16* amplicons (Fig 2B) and a slightly upward parabolic shape was obtained for *MLH1* (Fig 2C). The downward parabolic pattern implies preferential amplification of unmethylated templates relative to methylated templates (bias value, b = 0.70). Preferential amplification of unmethylated templates may lead to an underestimation of DNA methylation of the *P16* gene. The upward parabolic standard curve for *MLH1* could be interpreted in the opposite manner, i.e., it reflects preferential amplification of the methylated templates (b = 1.47) and results in an overestimation of methylation. The distinct bias patterns obtained from the melting analyses were confirmed by amplicon sequencing. Although NGS-based DNA methylation analysis is expensive and requires special expertise for data analysis, the method provides direct numerical information regarding the molecular composition of methylated and unmethylated DNA in a sample [17]. NGS reads for each amplicon were assigned either as unmethylated or methylated according to alignments to database comprising 0%-methylated and 100%-methylated sequences (S5 Table). Then, the read counts for unmethylated and methylated amplicons were calculated to estimate methylation levels, which were plotted as a standard curve. Bias patterns of standard curves from the melting analysis were reproduced in the standard curves from amplicon sequencing; linear, downward parabolic, and upward parabolic patterns for *P14*, *P16*, and *MLH1*, respectively (the rightmost panels in Fig 2). The reproduction of bias patterns across different post-PCR measurement platforms indicates that they were introduced during PCR processes, but not in post-PCR analyses. It is also noteworthy that the curvedness of the parabolic standard curves for *P16* and *MLH1* were more evident in the NGS results (b = 0.70 vs. 0.36 for *P16* and b = 1.47 vs. 2.53 for *MLH1*), while the linearity was maintained for *P14* (b = 1.02 vs. 1.05). The increased curvedness in NGS-based results was attributed to the 7 additional PCR cycles for attachment of adaptors and index sequences to amplicons for preparation of NGS libraries. This explanation is consistent with the inference that observed biases were introduced during PCR steps.

**Fig 3:**
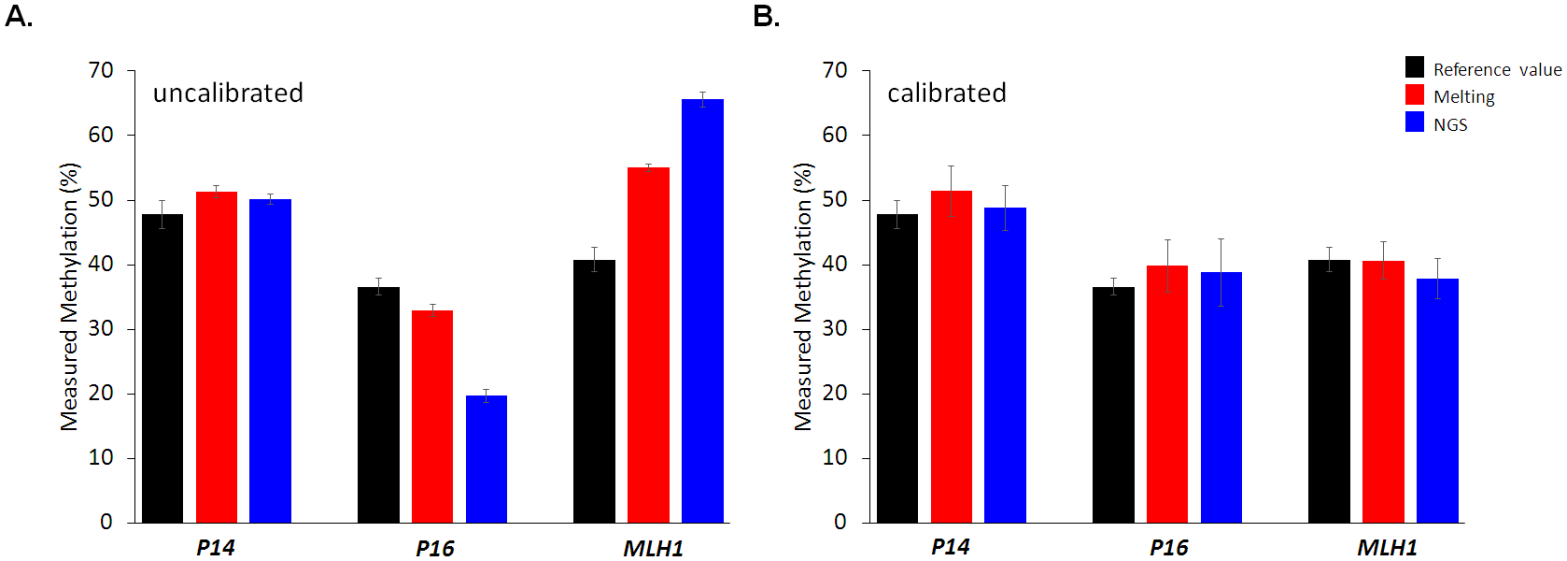
Calibration of analytical biases and accurate measurements of gene methylation of in-lab unknown samples. Raw results without calibration (**A**) and calibrated results using the template-type reference materials (**B**) are comparatively shown. Underestimating raw results for *P16* from both melting- and NGS-based analyses were successfully calibrated to correctly estimate the reference value of unknown sample. Overestimations for *MLH1* were also successfully calibrated. Results for *P14* with no detectable bias were correctly estimating the reference value without calibration. Error bars for reference values and calibrated values represent expanded uncertainties while those for uncalibrated values represent standard deviations.

The ultimate goal of the current measurement strategy using template-type reference materials is not only to precisely verify analytical biases, but also to properly calibrate those biases to achieve accurate measurements of gene methylation. To demonstrate the validity of our strategy, we prepared and tested three unknown samples by gravimetric mixing of unmethylated and *in vitro* methylated PCR products. The sample yields for *in vitro* methylation were greater than 95% for all three genes based on methyl cytosine contents estimated by dNMP analyses in CE (data not shown). The reference values for the in-lab unknown samples were also determined by CE-based measurements of methyl cytosine contents of the in-lab unknown samples before adding matrix DNA. Bisulfite-treated in-lab unknown samples with pre-defined reference values were analyzed in parallel with the working standards that were used as external calibrators. The concentration of working standards was matched with those of the unknown samples with respect to target genes, which will render target genes in samples and working standards to have similar amplification dynamics. Amplicons of unknown samples and working standards from exponential amplification phases were subjected to downstream melting-based and NGS-based DNA methylation analyses. Raw measured values either from melting analysis or NGS were calibrated by their respective standard curves from parallel working standards. We used a two-point calibration method in which unknown values were calculated by linear regression of the two closest neighboring points in the standard curve. The application of the two-point calibration method was inevitable because it was not easy to derive a mathematically well-defined fitting formula to the biased data for the *P16* and *MLH1* genes. Uncalibrated and calibrated values for each measurement method are compared in Fig 3. The uncalibrated values for *P16* were underestimating the reference value of unknown sample while values for *MLH1* were overestimating (Fig 3A). These results are consistent with the conclusion from Fig 2 that underestimation for *P16* and overestimation for *MLH1* will be obtained unless PCR biases are appropriately calibrated. Values for the bias-free *P14* were correctly estimating the reference value of unknown sample even without calibration as was also expected from Fig 2A. Contrastingly, calibrated values for all three genes were in good agreement with the reference values, which demonstrated the validity of our calibration strategy and template-type reference materials (Fig 3B). Calibration using our working standards resulted in correct measurements for both the melting and NGS-based analyses. These results indicated that biases that result from the PCR process and post-PCR analyses can be comprehensively calibrated using the current working standards, enabling accurate measurements of DNA methylation, irrespective of target gene and analytical platform.

It should be noted that the error bars for uncalibrated and calibrated values did not exhibit the same measurement characteristics. The error bars for uncalibrated values simply represent the coefficient of variation (CV) for repeated measurements, while those for calibrated values represent expanded uncertainty [33]. Measurement uncertainty was calculated by combining the CV for repeated measurements and uncertainty for calibrators, and then multiplying with a coverage factor of 2 for statistical expansion of measurement uncertainty. It is important to note that the smaller error bars for uncalibrated values do not mean accuracy and reliability of those measurement practices. They are small not because they were reliable and accurate but because they did not take essential components into account in calculation of measurement uncertainty.

**Fig 4:**
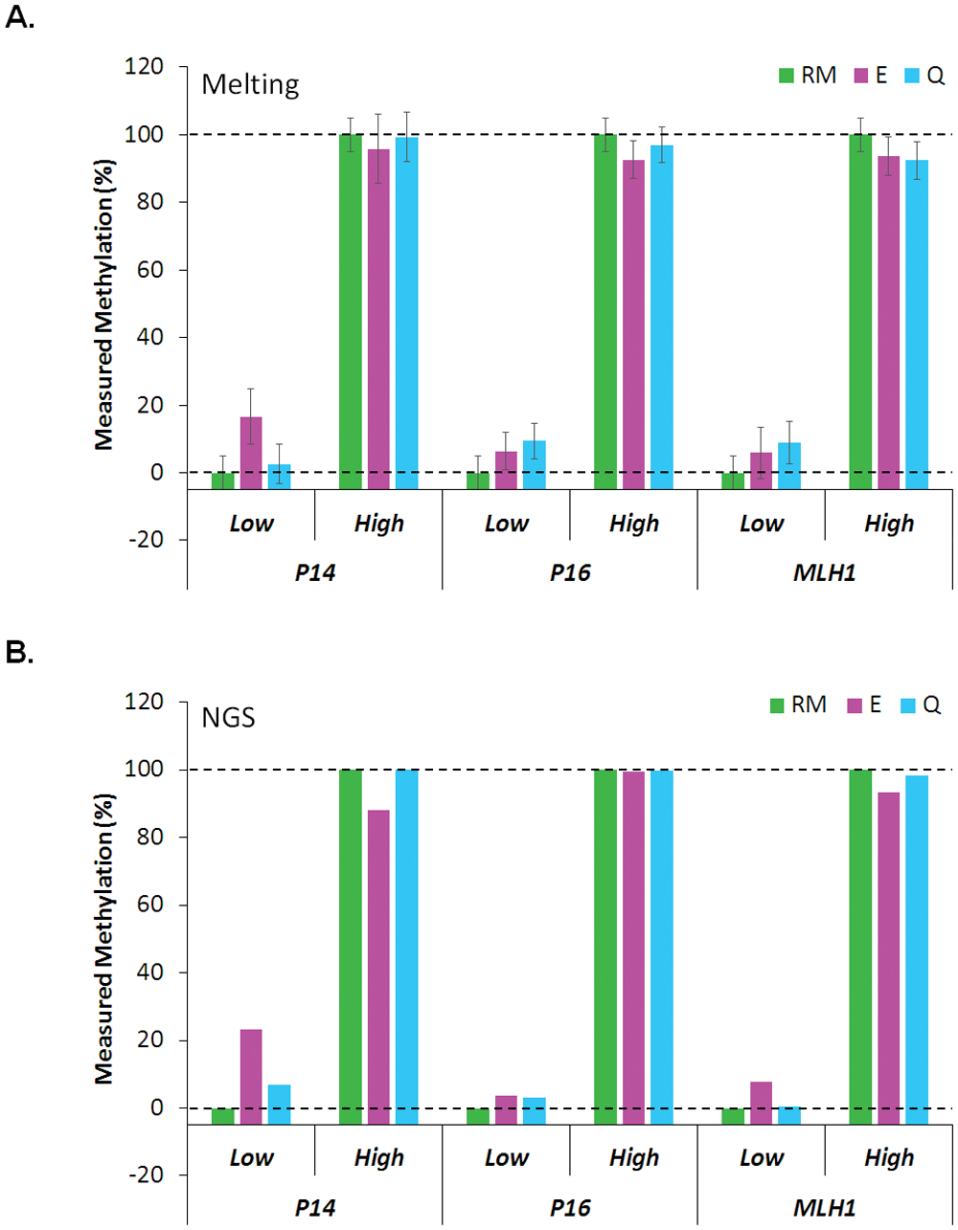
Comparative validation of methylation controls from commercial sources with the synthetic reference materials. Methylation levels of commercial DNA methylation controls and synthetic reference materials were measured by melting- (**A**) and NGS-based analyses (**B**).

The suggested reference materials enabled accurate and reliable quantification of DNA methylation as was demonstrated in this study. Nonetheless, we admit that the synthetic reference materials are not providing a perfect calibration system for wide and general uses but have a considerable weakness. The synthetic reference materials should be prepared gene-by-gene since a synthetic DNA would only represent a reference sequence of one methylation status of a gene. Gene- and methylation status-specific natures of the reference materials will result in higher costs and longer times for preparation. We consider the costs and times are inevitable tradeoffs for provision of accurate and reliable references for DNA methylation. There are several commercial DNA methylation control materials that were prepared based on enzymatic demethylation and in vitro methylation [26]. However, those control materials were not thoroughly validated if methylation states of specific genes are really in their guaranteed ranges, i.e. under 5% and over 85% for low and high methylation controls, respectively. To contrast the accuracy and reliability of our reference materials, we compared DNA methylation states of *P14*, *P16* and *MLH1* genes among two commercial controls and our synthetic references (Fig 4). Methylation levels were measured by both melting (Fig 4A) and NGS (Fig 4B) analyses. Methylation levels of commercial low methylation controls were measured to be 0.5–23.3% (average 7.9%) depending on genes and makes while the values from our M0 reference materials were stably around 0% (average 2.0%). The measured values were maintained in both melting and NGS analyses. The results indicate that not all genes in the commercial low methylation controls were within the guaranteed ranges possibly due to incomplete demethylation. Contrarily, very small differences were observed between DNA methylation values from commercial high methylation controls and our M100 references (average 95.8% vs. 98.3%). Based on these results, we concluded that our synthetic reference materials provide genuine 0% and 100% DNA methylation references while commercial DNA methylation controls could not always do that depending on genes and makes. Then, we regard the relatively higher cost for preparation of the synthetic reference materials is an acceptable tradeoff in pursuing accuracy and reliability of DNA methylation analyses.

In this paper, we suggested a calibration concept to use of template-type reference materials to facilitate accurate measurement of gene methylation. The proposed reference materials are characterized by two distinct properties. First, they include a pair of bisulfite-converted sequences that represent exactly 0%- and 100%-methylation status of a gene. Second, they are in discrete plasmid constructs that could be easily quantified and mixed as working standards. We demonstrated that various biases, either from PCR or post-PCR measurements, can be precisely verified using our reference materials. In addition, those biases were successfully calibrated leading to accurate and consistent measurements of DNA methylation, independent of target genes, PCR conditions, and post-PCR analytical platforms. Based on these results, we expect that the suggested reference materials could provide an accurate, easily reproducible, and widely applicable foundation for establishment of a standardized system to measure DNA methylation of CpG cytosines.

## Supporting Information

S1 Data Alignments of synthetic sequences. (DOCX)

S1 Table Primers and probes for relative quantification of plasmids. (DOCX)

S2 Table Primers for amplification of promoter regions for preparation of in-lab unknown samples. (DOCX)

S3 Table Primers for amplification of target loci for DNA methylation analyses. (DOCX)

S4 Table Relative quantities of a pair of reference plasmids for each gene. Results from qPCR experiments are represented as relative quantities of M0 to M100 plasmids. *u_std represents ‘standard uncertainty’ which is conventionally referred as ‘standard error of the mean’.
(DOCX)

S5 Table Results of amplicon sequencing of working standards and in-lab unknown samples for quantitative DNA methylation analysis. Reads were sorted and counted based on better matches either to M0 or M100 sequences. U_1, U_2 and U_3: triplicate measurements of in-lab unknown samples.
(DOCX)

S1 Fig Agarose gel image of gene-specific amplicons for DNA methylation analysis. Sizes of amplicons were 205-, 269- and 199-bp for *P14*, *P16* and *MLH1*, respectively. Amplicons with correct sizes from M0 and M100 controls were identified while no amplicons from no-template controls were detected.
(DOCX)

S2 Fig Restriction enzyme digestion patterns of *P14* amplicons. The methylated amplicon of *P14* represents an EcoRI site while the unmethylated amplicon does not. EcoR1-digestion patterns of amplicons from four selected working standards are shown.
(DOCX)

S3 Fig Maintenance of melting profiles before and after PCR purification. *P14* amplicons were analyzed by melting analyses before and after column purification. Melting profiles and their standard curves are maintained before and after purification.
(DOCX)
