# Supplementary material for: Reference Materials for Calibration of Analytical Biases in Quantification of DNA Methylation
Source: PLoS One. 2015 Sep 14;10(9):e0137006. doi: 10.1371/journal.pone.0137006 (PMC4569303; doi:10.1371/journal.pone.0137006)
Supplement: S1 Data — (DOCX) [file pone.0137006.s001.docx]

**S1 Data.**

**P14** (NC_000009.12; 21994978 – 21995241)

100% TAATTTAGTTTGAAGAATGGAAGATTTTCGACGAGGGGAGTTAGGAATAAAATAAGGGGA

0% TAATTTAGTTTGAAGAATGGAAGATTTTTGATGAGGGGAGTTAGGAATAAAATAAGGGGA

100% ATAGGGGAGCGGGGACGCGAGTAGTATTAGAATTCGCGGGAGCGCGGTTGTTTTTGGTAG

0% ATAGGGGAGTGGGGATGTGAGTAGTATTAGAATTTGTGGGAGTGTGGTTGTTTTTGGTAG

100% GGTCGTGTTAGGTGACGGATGTAGTTAGGGGGCGAGTTGTTTGGAGTTGCGTTTTAGGCG

0% GGTTGTGTTAGGTGATGGATGTAGTTAGGGGGTGAGTTGTTTGGAGTTGTGTTTTAGGTG

100% TTCGGTTTTTGGGTCGTTATCGCGGGGCGTTCGCGTTGAGGGTGGGAAGATGGTGGTGGG

0% TTTGGTTTTTGGGTTGTTATTGTGGGGTGTTTGTGTTGAGGGTGGGAAGATGGTGGTGGG

100% GGTGGGGGCGTATATAGGGCGGGAA

0% GGTGGGGGTGTATATAGGGTGGGAA

**P16** (NC_000009.12; 21974629 – 21975018)

100% TTTTAGAGGATTTGAGGGATAGGGTCGGAGGGGGTTTTTTCGTTAGTATCGGAGGAAGAA

0% TTTTAGAGGATTTGAGGGATAGGGTTGGAGGGGGTTTTTTTGTTAGTATTGGAGGAAGAA

100% AGAGGAGGGGTTGGTTGGTTATTAGAGGGTGGGGCGGATCGCGTGCGTTCGGCGGTTGCG

0% AGAGGAGGGGTTGGTTGGTTATTAGAGGGTGGGGTGGATTGTGTGTGTTTGGTGGTTGTG

100% GAGAGGGGGAGAGTAGGTAGCGGGCGGCGGGGAGTAGTATGGAGTCGGCGGCGGGGAGTA

0% GAGAGGGGGAGAGTAGGTAGTGGGTGGTGGGGAGTAGTATGGAGTTGGTGGTGGGGAGTA

100% GTATGGAGTTTTCGGTTGATTGGTTGGTTACGGTCGCGGTTCGGGGTCGGGTAGAGGAGG

0% GTATGGAGTTTTTGGTTGATTGGTTGGTTATGGTTGTGGTTTGGGGTTGGGTAGAGGAGG

100% TGCGGGCGTTGTTGGAGGCGGGGGCGTTGTTTAACGTATCGAATAGTTACGGTCGGAGGT

0% TGTGGGTGTTGTTGGAGGTGGGGGTGTTGTTTAATGTATTGAATAGTTATGGTTGGAGGT

100% CGATTTAGGTGGGTAGAGGGTTTGTAGCGGGAGTAGGGGATGGCGGGCGATTTTGGAGGA

0% TGATTTAGGTGGGTAGAGGGTTTGTAGTGGGAGTAGGGGATGGTGGGTGATTTTGGAGGA

100% CGAAGTTTGTAGGGGAATTGGAATTAGGTA

0% TGAAGTTTGTAGGGGAATTGGAATTAGGTA

**MLH1** MLH1 (NC_000003.12; 36992797 – 36993147)

100% TTTAGGAGTGAAGGAGGTTACGGGTAAGTCGTTTTGACGTAGACGTTTTATTAGGGTCGC

0% TTTAGGAGTGAAGGAGGTTATGGGTAAGTTGTTTTGATGTAGATGTTTTATTAGGGTTGT

100% GCGTTCGTCGTTCGTTATATATCGTTCGTAGTATTCGTGTTTAGTTTCGTAGTGGCGTTT

0% GTGTTTGTTGTTTGTTATATATTGTTTGTAGTATTTGTGTTTAGTTTTGTAGTGGTGTTT

100% GACGTCGCGTTCGCGGGTAGTTACGATGAGGCGGCGATAGATTAGGTATAGGGTTTTATC

0% GATGTTGTGTTTGTGGGTAGTTATGATGAGGTGGTGATAGATTAGGTATAGGGTTTTATT

100% GTTTTTCGGAGGTTTTATTATTAAATAACGTTGGGTTTATTCGGGTCGGAAAATTAGAGT

0% GTTTTTTGGAGGTTTTATTATTAAATAATGTTGGGTTTATTTGGGTTGGAAAATTAGAGT

100% TTCGTCGATTTTTATTTTGTTTTTTTTGGGCGTTATTTATATTTTGCGGGAGGTTATAAG

0% TTTGTTGATTTTTATTTTGTTTTTTTTGGGTGTTATTTATATTTTGTGGGAGGTTATAAG

100% AGTAGGGTTAACGTTAGAAAGGTCGTAAGGGGAGAGGAGGAGTTTGAGAAG

0% AGTAGGGTTAATGTTAGAAAGGTTGTAAGGGGAGAGGAGGAGTTTGAGAAG
