# Supplementary material for: Reference Materials for Calibration of Analytical Biases in Quantification of DNA Methylation
Source: PLoS One. 2015 Sep 14;10(9):e0137006. doi: 10.1371/journal.pone.0137006 (PMC4569303; doi:10.1371/journal.pone.0137006)
Supplement: S1 Table — (DOCX) [file pone.0137006.s002.docx]

**S1 Table.**

| Target locus | Sequence |
| --- | --- |
| *bla*-1 | Forward: 5’- AAGTTGGCGGCATTATC -3’  Reverse: 5’- GCTATGTGGCGCGGTATTAT -3’  Probe: 5’- FAM-ATCCGTAAGATGCTTTTCTGTGAC-BHQ1 -3’ |
| *bla*-2 | Forward: 5’- TTGCCGGGAAGCTAGAGTAA -3’  Reverse: 5’- AAGCCATACCAAACGACGAG -3’  Probe: 5’- FAM-CGCCAGTTAATAGTTTGCGC-BHQ1 -3’ |
| *ori*-1 | Forward: 5’- GGCGCTTTCTCATAGCTCAC -3’  Reverse: 5’- AGTCGTGTCTTACCGGGTTG -3’  Probe: 5’- FAM-CAA GCT GGG CTG TGT GCA CGA A-BHQ1 -3’ |
| *ori*-2 | Forward: 5’- GCCTACATACCTCGCTCTGC -3’  Reverse: 5’- AGTTCGGTGTAGGTCGTTCG -3’  Probe: 5’- FAM-CAAGCTGGGCTGTGTGCACGAA-BHQ1 -3’ |
