# Supplementary material for: Reference Materials for Calibration of Analytical Biases in Quantification of DNA Methylation
Source: PLoS One. 2015 Sep 14;10(9):e0137006. doi: 10.1371/journal.pone.0137006 (PMC4569303; doi:10.1371/journal.pone.0137006)
Supplement: S2 Table — (DOCX) [file pone.0137006.s003.docx]

**S2 Table.**

| Target gene | Sequence |
| --- | --- |
| *P14*  (NC_000009.12; 21994642 – 21995215) | Forward: 5’- CACAAAGGACTCGGTGCTTG -3’  Reverse: 5’- CCACCACCATCTTCCCA -3’ |
| *P16*  (NC_000009.12; 21974512 – 21975101) | Forward: 5’- AGACCCAACCTGGGGCGACT -3’  Reverse: 5’- CGTCGCCAGGAGGAGGTCTG -3’ |
| *MLH1*  (NC_000003.12; 36992695 – 36993672) | Forward: 5’- AACACCTCCATGCACTGGTA -3’  Reverse: 5’- CCTCCGTACCAGTTCTCAA -3’ |
