# Supplementary material for: Reference Materials for Calibration of Analytical Biases in Quantification of DNA Methylation
Source: PLoS One. 2015 Sep 14;10(9):e0137006. doi: 10.1371/journal.pone.0137006 (PMC4569303; doi:10.1371/journal.pone.0137006)
Supplement: S3 Table — (DOCX) [file pone.0137006.s004.docx]

**S3 Table.**

| Target gene | Sequence |
| --- | --- |
| *P14*_BS | Forward: 5’- TTATTTAGTTTGAAGAATGGAAGAT -3’  Reverse: 5’- ACCACCATCTTCCCACCCTCAA -3’ |
| *P16*_BS | Forward: 5’- TTTAGAGGATTTGAGGG -3’  Reverse: 5’- TACCTAATTCCAATTCCCCTACAAAC -3’ |
| *MLH1*_BS | Forward: 5’- TATGTATTGGTATATAAAGTTT -3’  Reverse: 5’- TTAACCCTACTCTTATAACCTCC -3’ |
