# Supplementary material for: Reference Materials for Calibration of Analytical Biases in Quantification of DNA Methylation
Source: PLoS One. 2015 Sep 14;10(9):e0137006. doi: 10.1371/journal.pone.0137006 (PMC4569303; doi:10.1371/journal.pone.0137006)
Supplement: S4 Table — Results from qPCR experiments are represented as relative quantities of M0 to M100 plasmids. *u_std represents ‘standard uncertainty’ which is conventionally referred as ‘standard error of the mean’. (DOCX) [file pone.0137006.s005.docx]

**S4 Table**.

| qPCR | *P14* | | *P16* | | *MLH1* | |
| --- | --- | --- | --- | --- | --- | --- |
|  | M0 | M100 | M0 | M100 | M0 | M100 |
| bla-1 | 100.0 | 92.0 | 100.0 | 65.6 | 100.0 | 82.0 |
| bla-2 | 100.0 | 105.2 | 100.0 | 60.7 | 100.0 | 77.0 |
| ori-1 | 100.0 | 101.3 | 100.0 | 65.1 | 100.0 | 76.6 |
| ori-2 | 100.0 | 95.2 | 100.0 | 74.5 | 100.0 | 70.3 |
| Mean | 100.0 | 98.4 | 100.0 | 66.5 | 100.0 | 76.5 |
| u_std* (%) | 3.0 | 3.0 | 4.3 | 4.3 | 3.1 | 3.1 |
