# Supplementary material for: Reference Materials for Calibration of Analytical Biases in Quantification of DNA Methylation
Source: PLoS One. 2015 Sep 14;10(9):e0137006. doi: 10.1371/journal.pone.0137006 (PMC4569303; doi:10.1371/journal.pone.0137006)
Supplement: S5 Table — Reads were sorted and counted based on better matches either to M0 or M100 sequences. U_1, U_2 and U_3: triplicate measurements of in-lab unknown samples. (DOCX) [file pone.0137006.s006.docx]

**S5 Table.**

| ***P14*** | | | ***P16*** | | | ***MLH1*** | | |
| --- | --- | --- | --- | --- | --- | --- | --- | --- |
| Prepared (%) | Read counts (M/U) | Measured (%) | Prepared (%) | Read counts (M/U) | Measured (%) | Prepared (%) | Read counts (M/U) | Measured (%) |
| 0.0 | 56 / 86051 | 0.1 | 0.0 | 21 / 65243 | 0 | 0.0 | 324 / 27269 | 1.2 |
| 9.8 | 2253 / 18142 | 11.0 | 6.9 | 799 / 20583 | 3.7 | 7.9 | 20080 / 43978 | 31.3 |
| 19.7 | 4669 / 18194 | 20.4 | 14.3 | 2284 / 21621 | 9.6 | 16.1 | 21737 / 25693 | 45.8 |
| 29.6 | 11560 / 25525 | 31.2 | 22.3 | 4542 / 31472 | 12.6 | 24.8 | 50485 / 45133 | 52.8 |
| 39.5 | 25807 / 35051 | 42.4 | 30.9 | 5846 / 30410 | 16.1 | 33.9 | 36712 / 23592 | 60.9 |
| 49.5 | 19981 / 19061 | 51.2 | 40.1 | 4615 / 17350 | 21 | 43.5 | 53699 / 20409 | 72.5 |
| 59.5 | 20116 / 13125 | 60.5 | 50.1 | 2976 / 8230 | 26.6 | 53.6 | 39779 / 13620 | 74.5 |
| 69.6 | 13825 / 5808 | 70.4 | 61.0 | 4746 / 9565 | 33.2 | 64.2 | 80604 / 18783 | 81.1 |
| 79.7 | 33260 / 7998 | 80.6 | 72.8 | 7306 / 8466 | 46.3 | 75.5 | 71527 / 12688 | 84.9 |
| 89.8 | 16040 / 1769 | 90.1 | 85.8 | 7066 / 4080 | 63.4 | 87.4 | 65243 / 5868 | 91.7 |
| 100.0 | 67592 / 98 | 99.9 | 100.0 | 12755 / 54 | 99.6 | 100.0 | 54399 / 251 | 99.5 |
| **U_1** | 72460 / 70229 | 50.8 | **U_1** | 9459 / 35936 | 20.8 | **U_1** | 143980 / 72330 | 66.6 |
| **U_2** | 96793 / 99948 | 49.2 | **U_2** | 10298 / 43107 | 19.3 | **U_2** | 225387 / 116671 | 65.9 |
| **U_3** | 31330 / 30813 | 50.4 | **U_3** | 6425 / 27612 | 18.9 | **U_3** | 296550 / 165011 | 64.2 |
