# Supplementary figures and images for: Reference Materials for Calibration of Analytical Biases in Quantification of DNA Methylation
Source: PLoS One. 2015 Sep 14;10(9):e0137006. doi: 10.1371/journal.pone.0137006 (PMC4569303; doi:10.1371/journal.pone.0137006)

**S1 Fig.**


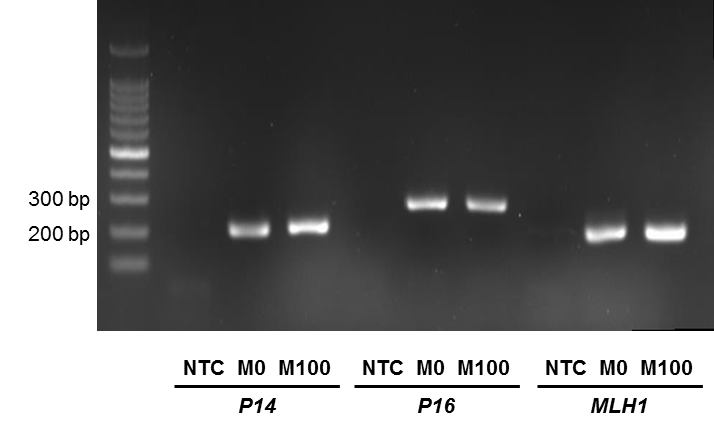

Supplement: S1 Fig — Sizes of amplicons were 205-, 269- and 199-bp for P14, P16 and MLH1, respectively. Amplicons with correct sizes from M0 and M100 controls were identified while no amplicons from no-template controls were detected. (DOCX) [file pone.0137006.s007.docx]

**S2 Fig.**


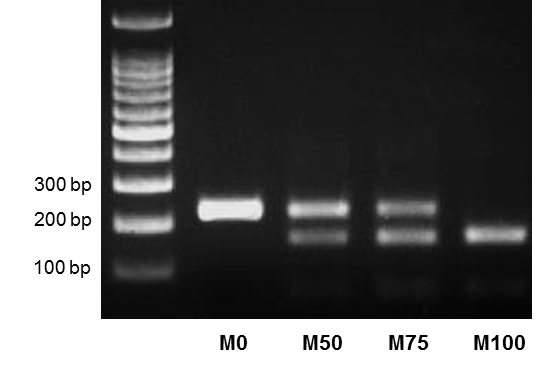

Supplement: S2 Fig — The methylated amplicon of P14 represents an EcoRI site while the unmethylated amplicon does not. EcoR1-digestion patterns of amplicons from four selected working standards are shown. (DOCX) [file pone.0137006.s008.docx]

**S3 Fig.**


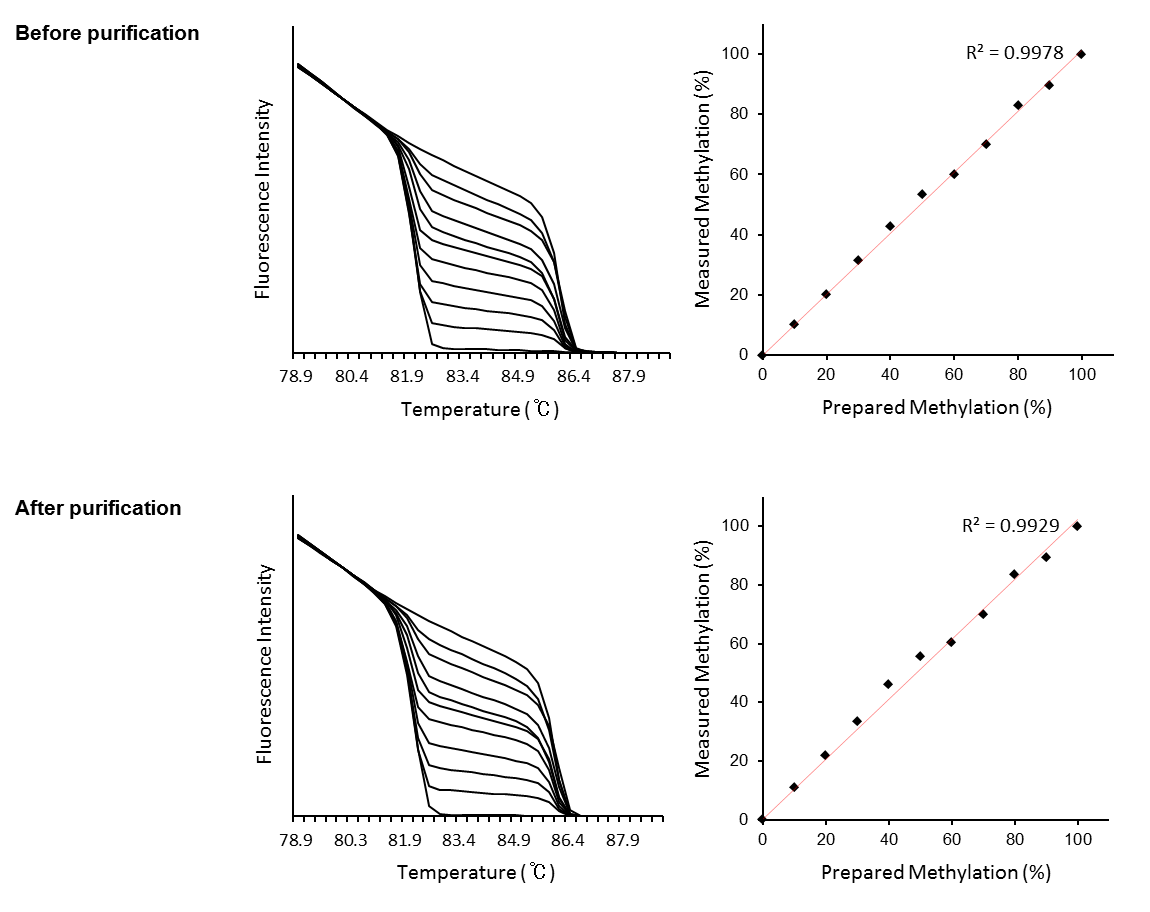

Supplement: S3 Fig — P14 amplicons were analyzed by melting analyses before and after column purification. Melting profiles and their standard curves are maintained before and after purification. (DOCX) [file pone.0137006.s009.docx]
